# Increased circulating regulatory T cells and decreased follicular T helper cells are associated with colorectal carcinogenesis

**DOI:** 10.3389/fimmu.2024.1287632

**Published:** 2024-01-26

**Authors:** Qiao Meng, Yang Zhao, Miao Xu, Pingzhang Wang, Jun Li, Rongli Cui, Weiwei Fu, Shigang Ding

**Affiliations:** ^1^ Department of Gastroenterology, Peking University Third Hospital, Beijing, China; ^2^ Beijing Key Laboratory for Helicobacter Pylori Infection and Upper Gastrointestinal Diseases, Beijing, China; ^3^ Department of Laboratory Medicine, Peking University Third Hospital, Beijing, China; ^4^ Broad Institute of Harvard and Massachusetts Institute of Technology, Cambridge, MA, United States; ^5^ Department of Immunology, School of Basic Medical Sciences, Peking University Health Science Center, NHC Key Laboratory of Medical Immunology (Peking University), Beijing, China

**Keywords:** colorectal cancer, adenoma, hyperplastic polyps, T helper cell, innate lymphoid cells

## Abstract

**Objective:**

Colorectal cancer (CRC) is the third most prevalent cancer worldwide and is associated with high morbidity and mortality rates. Colorectal carcinogenesis occurs via the conventional adenoma-to-carcinoma and serrated pathways. Conventional T helper (Th) and innate lymphoid cells (ILCs) play vital roles in maintaining intestinal homeostasis. However, the contribution of these two major lymphoid cell populations and their associated cytokines to CRC development is unclear. Therefore, we aimed to analyze peripheral lymphocyte profiles during colorectal carcinogenesis.

**Methods:**

We collected 86 blood samples concurrently, and pathologists confirmed the presence of various pathological conditions (i.e., HPs, adenoma, and carcinoma) using hematoxylin and eosin staining. Ten healthy donors were recruited as healthy controls (HCs) from the physical examination center. We performed flow cytometry on peripheral blood mononuclear cells collected from patients with various pathological conditions and the HCs, and cytokines (interleukin-2, interleukin-4, interleukin-5, interleukin-13, interleukin-17A, interleukin-17F, interleukin-22, interferon-γ, and tumor necrosis factor-α) were quantified. We also analyzed the published single-cell RNA sequence data derived from tissue samples from different stages of colorectal carcinogenesis.

**Results:**

The cytokine response in peripheral CD4^+^ T cells was upregulated during the carcinoma process. The frequency of peripheral regulatory T cells (Tregs) increased in the adenoma and carcinoma stages. While the T follicular helper (Tfh) cell proportion was downregulated in the adenoma and carcinoma processes. Thus, Th cell subsets, especially Tregs and Tfh cells, were involved in colonic diseases. Moreover, the immunological profile characteristics in the HPs were clarified.

**Conclusion:**

We comprehensively analyzed circulating ILCs and adaptive T-cell lymphocyte subtypes in colorectal carcinoma progression. Our results show the immunological profile characteristics and support the involvement of Th subsets, especially Treg and Tfh cell populations, in colonic diseases. These findings significantly enhance our understanding of the immune mechanisms underlying CRC and its precancerous lesions. Further investigation of the Treg and Tfh cells’ function in colorectal disease development will provide potential therapeutic targets for monitoring and preventing CRC development.

## Introduction

Colorectal cancer (CRC) is the third most prevalent cancer worldwide, with over 1.9 million new CRC cases and 0.93 million deaths estimated in 2020 (1). Although the death rate slightly decreased (2), CRC mortality still ranks second among all cancer-related death cases worldwide (3). Colorectal carcinogenesis can occur via the conventional adenoma-to-carcinoma and serrated pathways. The typical adenoma–carcinoma sequence is an established model for sporadic CRC development and causes approximately 60%–85% of colonic malignancies. Precursor lesions that display tubular, tubulovillous, or villous adenoma histology develop into low- or high-grade adenomas and CRC (4). However, the mechanisms underlying CRC onset and progression are not fully understood. Furthermore, over 15% of CRC cases arise through the serrated pathway (5). Hyperplastic polyps (HPs), classified as serrated lesions, have shown that cells only exhibit minimal cytological atypia in the upper two-thirds of the crypts (6). HPs often coexist with adenoma and adenocarcinoma and have distinct biological features in CRC (7, 8). Therefore, the characteristics of HPs must be clarified.

The tumor immune microenvironment plays a crucial role in CRC development (9), determines the durable response to immunotherapy, and may be a predictive biomarker (10–12). Progression from precancerous lesions to malignant CRC depends on a complex immune pathway involving activated T lymphocytes and cytotoxic cytokine production. Previous cancer immunological studies have primarily focused on CD8^+^ T cells. However, recent studies highlighted the importance of CD4^+^ T cells, considering that CD4^+^ T cells are central coordinators of innate and antigen-specific immune responses (13). Innate lymphoid cells (ILCs) are heterogeneous immune cells with no antigen-specific receptors that produce cytokines similar to CD4^+^ T helper (Th) subsets (14, 15). Conventional Th cells and ILCs play critical roles in maintaining intestinal homeostasis. However, Cui et al. have found that interleukin (IL)-17A plays important roles along the colorectal adenoma–carcinoma sequence (16–18). However, how these two major lymphoid cell populations and their related cytokines are involved in CRC development has not been determined systematically.

Recently, Zheng et al. dissected dynamic alterations in cell populations in the normal adenoma–carcinoma sequence using single-cell RNA-sequencing technology (19). Furthermore, using single-cell transcriptomic analysis, Chen et al. demonstrated the different immune microenvironmental features of conventional adenomas and serrated polyps (20). The occurrence and development of tumors are accompanied by systemic immune disturbance and peripheral immune cell alterations (21–24). Individual immunity is coordinated across tissues, and the colonic antitumor immune response depends on continuous communication with the peripheral blood (25, 26). Our previous study demonstrated peripheral adaptive and innate lymphocyte changes during human gastric cancer development (27). Considering the easily obtainable and non-invasive characteristics of peripheral blood samples, exploring changes in peripheral immune profiles is crucial and may help discover key functional cell subpopulations during CRC carcinogenesis. Therefore, novel target cells can be provided for the early screening of CRC patients.

This study comprehensively analyzed peripheral lymphocyte profiles in HPs and conventional adenoma–carcinoma sequences.

## Materials and methods

### Patients and controls

Patients were admitted to Peking University Third Hospital between December 2021 and August 2022. Eighty-six patients diagnosed with HPs (*n* = 10), adenomas (grade I, *n* = 19; grade II, *n* = 19; grade III, *n* = 19), or adenocarcinomas (*n* = 19) were enrolled. All patients were diagnosed based on histological examination results. Patients with active systemic infections or autoimmune diseases were excluded. Ten healthy donors were recruited as healthy controls (HCs) from the physical examination center. Two milliliters of the remaining blood samples from routine complete blood count tests were collected as peripheral blood samples for the experiment. The Medical Science Research Ethics Committee of Peking University Third Hospital approved this study (2022 YLS No. 554-01).

### Cell isolation and flow cytometry analysis

Peripheral blood mononuclear cells (PBMCs) were isolated from heparinized peripheral blood through Ficoll-Hypaque gradients as previously described (28). The collected PBMCs were resuspended and cryopreserved with freezing media, including 10% dimethylsulfoxide (DMSO) and 90% fetal bovine serum, and then stored in a freezing container at −80°C. For flow cytometry analysis, the cryopreserved PBMCs were placed in a 37°C water bath for rapid thawing and then transferred into a 15-mL conical centrifuge tube filled with prewarmed RPMI 1640. After the washing step, the cells were resuspended in a culture medium and incubated at 37°C for 2 h before staining or stimulation.

For staining, the Fixable Viability Dye eFluor 506 (Thermo Fisher Scientific, Waltham, MA, USA) was stained firstly to gate live cells, followed by 30 min of surface antibody staining at 4°C. The antibodies used for flow cytometry are listed in **Supplementary Table 1**. For cytokine detection, PBMCs were stimulated for 4.5 h with 50 ng/mL of phorbol 12-myristate 13-acetate and 500 ng/mL of ionomycin (Sigma-Aldrich, St. Louis, MO, USA) in the presence of GolgiStop (BD/PMG) in a 37°C incubator before staining. Cells were fixed, permeabilized, and stained using the Cytofix/Cytoperm Kit (BD). Flow cytometry was performed using a FACSCanto instrument (BD Biosciences, San Jose, CA, USA) and analyzed using FlowJo software 10.8.1. The cell type annotation and gating strategy were performed as shown previously (27–29).

### Cytokine measurement

Cytokines [IL-2, IL-4, IL-5, IL-13, IL-17A, IL-17F, IL-22, interferon (IFN)-γ, and tumor necrosis factor (TNF)-α] in the plasma were measured using the LEGENDplex™ Multi-Analyte Flow Assay kit (BioLegend, San Diego, CA, USA) following the manufacturer’s protocol and Canto flow cytometry.

### Single-cell RNA sequence data analysis

A single-cell dataset derived from colorectal adenoma and carcinoma tissues was downloaded from the Gene Expression Omnibus database (GSE161277) (19). Standard analysis was performed using the R Seurat package (version 3.1.2) (29). Briefly, using the Read10X function to read the output results, the results were converted to Seurat objects using the CreateSeuratObject function. Each cell was normalized to 10,000 unique molecular identifier counts, and the top 2,000 highly variable genes (HVGs) were selected. After the data were scaled and centered, principal component analysis based on the HVGs was conducted. The uniform manifold approximation and projection method was used for dimensionality reduction in single-cell cluster visualization. Cell types were annotated according to marker gene expression, such as CD3 and CD4 for CD4^+^ T cells. In CD4^+^ T cells, dot plots were generated to show gene expression using the DotPlot function. The cell annotation strategy in DotPlot is according to the DICE database (https://dice-database.org/).

### Statistical analyses

Differences between groups were analyzed using a one-way ANOVA if the data followed a normal distribution. Otherwise, we used the non-parametric Kruskal–Wallis test. Analyses were conducted using GraphPad Prism 8.0 (GraphPad Software, San Diego, CA, USA). All statistical tests were two-tailed. A value of *P <*0.05 was considered significant.

## Results

### Cytokine profile analysis during colorectal carcinogenesis

**Figure 1:**
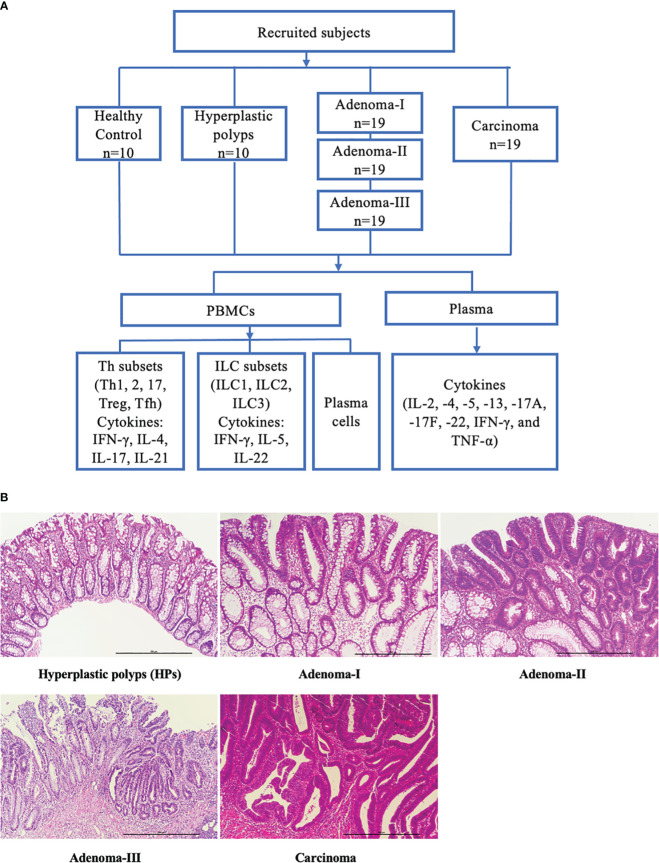
Study flowchart and pathologic diagnosis of enrolled patients. **(A)** Subdivision of enrolled subjects into health controls, hyperplastic polyps, adenoma (grades I, II, and III), and carcinoma. The peripheral blood samples were collected, and the key lymphocyte subpopulations in PBMCs and the plasma cytokines were analyzed. **(B)** Representative H&E-stained sections of a cascade of colonic lesions. Scale bar: 500 μm (×100 magnification).

To demonstrate the dynamic changes in the immune landscape during CRC development, we collected 86 blood samples to analyze the major lymphoid cell populations and their related cytokines (**Figure 1A**), and the pathological conditions were confirmed (**Figure 1B**). The clinical characteristics of the patients and HCs are shown in **Table 1**.

**Table 1:** Characteristics of enrolled patients with colonic lesions.

	Health control (*n* = 10)	Hyperplastic polyp (*n* = 10)	Tubular adenoma grade I (*n* = 19)	Tubular adenoma grade II (*n* = 19)	Tubular adenoma grade III (*n* = 19)	Colorectal carcinoma (*n* = 19)
Median age (years, range)	42 (39, 47)	61 (43, 68)	59 (30, 74)	64 (38, 77)	63 (42, 77)	67 (21, 90)
Gender						
Male	7 (70.0%)	8 (80.0%)	7 (36.8%)	7 (36.8%)	10 (52.6%)	15 (78.9%)
Female	3 (30.0%)	2 (20.0%)	12 (63.2%)	12 (63.2%)	9 (47.4%)	4 (21.1%)
Histological diagnosis	Untested	Hyperplastic polyp	Tubular adenoma grade I	Tubular adenoma grade II	Tubular adenoma grade III	Colorectal carcinoma

**Figure 2:**
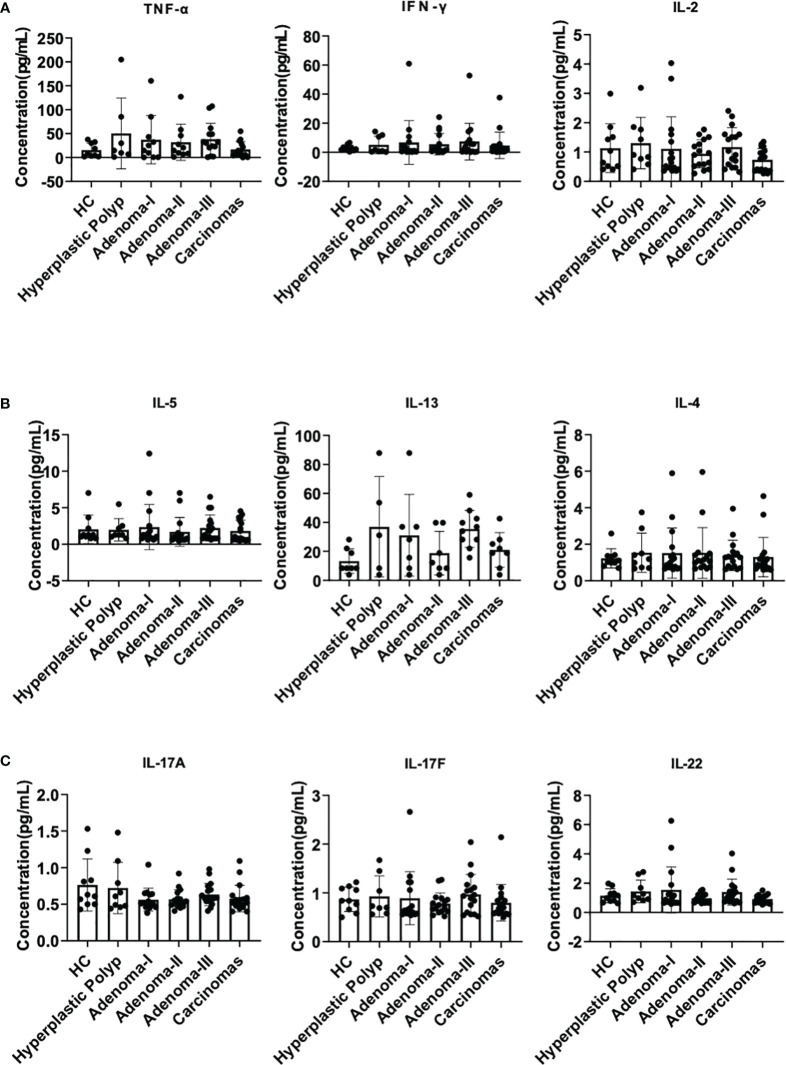
Plasma cytokine analysis during a cascade of colonic lesions. **(A)** The concentration of TNF-α, IFN-γ, and IL-2 in the plasma. **(B)** The concentration of IL-5, IL-13, and IL-4 in the plasma. **(C)** The concentration of IL-17A, 17F, and 22 in the plasma. Each dot represents one donor. HC, healthy control. Error bars represent the SEM. ANOVA or non-parametric test (Kruskal–Wallis test) as appropriate. All results are not significantly different. *n* = 10–19 in each group.

Firstly, we detected IL-2, IL-4, IL-5, IL-13, IL-17A, IL-17F, IL-22, IFN-γ, and TNF-α in the plasma to exclude the influence of infection or autoimmune disorders, and the standard curves were verified (**Supplementary Figure 1**). IL-2, IL-4, IL-5, IL-13, IL-17A, IL-17F, IL-22, IFN-γ, and TNF-α exhibited no apparent difference between each group, indicating the comparable inflammatory conditions between these samples (all *P* > 0.05) (**Figures 2A–C**). These results revealed that the plasma cytokine profile changed subtly, and their ability to indicate lesions was limited.

### The cytokine response in peripheral CD4^+^ T cells is upregulated during the carcinoma process

**Figure 3:**
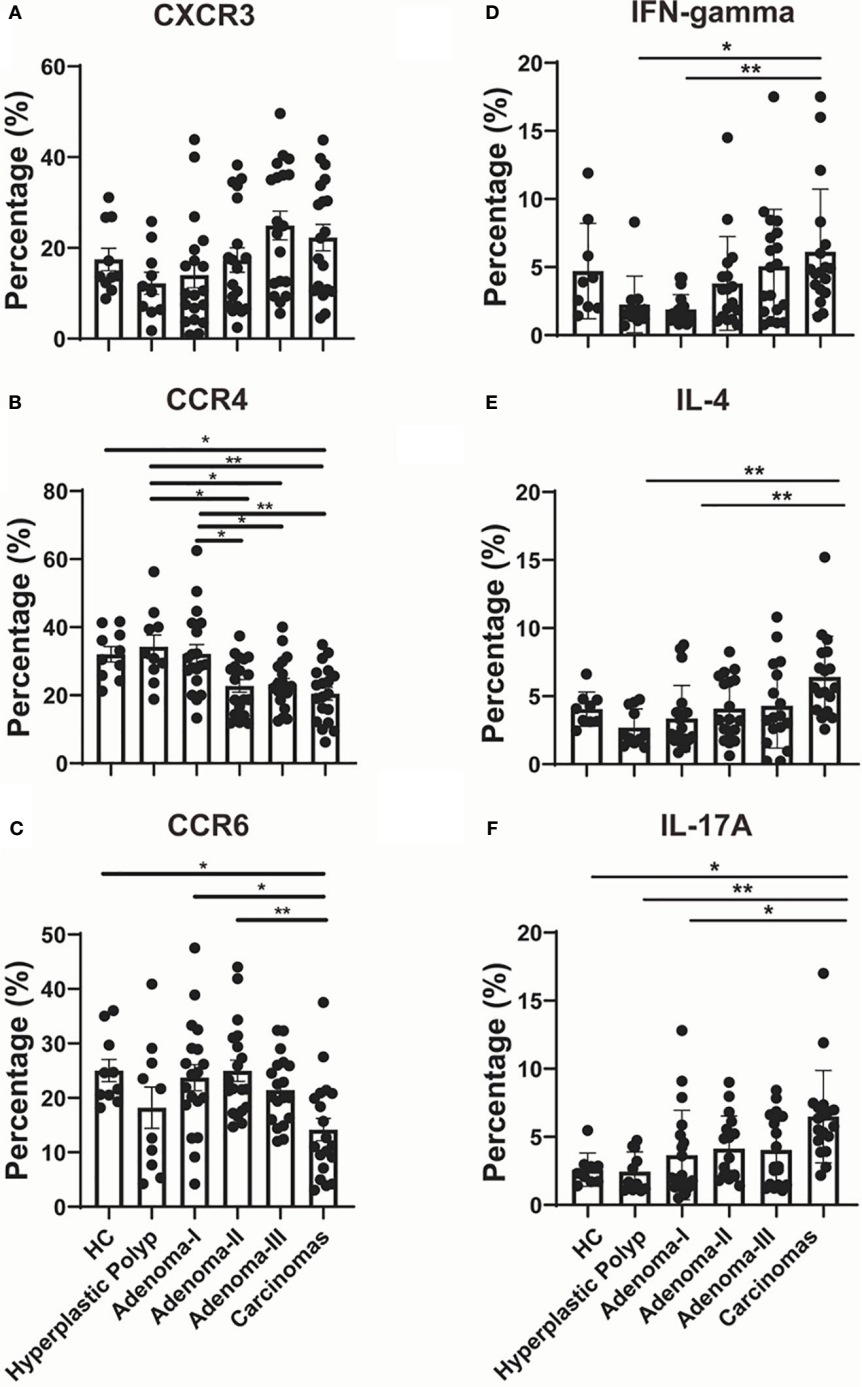
Th cell response analysis during a cascade of colonic lesions. **(A–C)** Flow cytometry quantification of the frequency of CXCR3^+^ **(A)**, CCR4^+^ **(B)**, and CCR6^+^ **(C)** cells in CD4^+^ T cells during a cascade of colonic lesions. **(D-F)** Flow cytometry quantification of the frequency of IFN-gamma^+^ **(D)**, IL-4^+^ **(E)**, and IL-17A^+^ **(F)** cells in CD4^+^ T cells during a cascade of colonic lesions. Each dot represents one donor. HC, healthy control. Error bars represent the SEM. *P < 0.05; **P < 0.01 (ANOVA or non-parametric test as appropriate). *n* = 10–19 in each group.

Firstly, we characterized the circulating Th cell subsets in the peripheral blood sample from healthy controls and patients with different colorectal lesions using surface markers. As shown in **Figure 3A**, CD4^+^CXCR3^+^ Th1 cells had no differences among the groups (**Figure 3A**). Compared with HPs, Th2 cells were decreased in adenoma grade II (31.6 vs. 24.7, *P* = 0.0222), adenoma grade III (31.6 *vs.* 22.2, *P* = 0.0313), and the CRC group (31.6 vs. 20.4, *P* = 0.0029) (**Figure 3B**). The percentage of Th2 cells was downregulated significantly from adenoma grade I to CRC (**Figure 3B**). As shown in **Figure 3C**, the CD4^+^CCR6^+^ Th17 cell proportion decreased in CRC when compared with the healthy control group (23.1 vs. 11.2, *P* = 0.0279) or adenoma grade I (24.3 vs. 11.2, *P* = 0.0163) or even adenoma grade II (21.7 vs. 11.2, *P* = 0.0042).

Moreover, we demonstrated the function of the peripheral Th cell subsets during carcinoma progression through key cytokine production analysis. IFN-gamma^+^ Th1 cells increased in CRC compared with those in HPs (1.61 vs. 4.73, *P* = 0.0267) (**Figure 3D**). In addition, the Th1 cell response was upregulated significantly from adenoma grade I to CRC (**Figure 3D**). As shown in **Figure 3E**, CD4^+^IL-4^+^ Th2 cells were increased in the CRC group when compared with those in HPs and adenoma grade I (2.11 vs. 5.63, *P* = 0.0027; 2.20 vs. 5.63, *P* = 0.0049). Furthermore, the proportion of CD4^+^IL-17A^+^ Th17 cells was investigated during CRC development. As shown in **Figure 3F**, Th17 cells increased in CRC when compared with the healthy control group (2.32 vs. 6.06, *P* = 0.0458) or HPs (1.68 vs. 6.06, *P* = 0.0014) or even adenoma grade I (1.99 vs. 6.06, *P* = 0.0130).

Furthermore, we analyzed published single-cell RNA sequence data (GSE161277) from tissue samples of different colorectal carcinogenesis stages to confirm the effector Th cell response during the adenoma–carcinoma sequence. As shown in **Supplementary Figure 2**, CD4^+^ T cells in tissue samples showed consistency only to the cytokine expression and not to the protein surface markers with that of the peripheral effector Th cell response. Th1 cells, identified by the *CXCR3* or *IFN-gamma* expression in the tissue-derived CD4^+^ T cells, were upregulated from adenoma to carcinoma (**Supplementary Figure 2**). Moreover, the upregulated Th2 cells, identified by the *CCR4* expression in the tissue-derived CD4^+^T cells, from adenoma to CRC were consistent with the peripheral CD4^+^IL-4^+^ Th2 response (**Supplementary Figure 2**). Lastly, we also detected increased Th17 populations, identified by the *CCR6* expression in the tissue-derived CD4^+^ T cells, in the CRC group when compared with the normal group, which was consistent with the peripheral CD4^+^IL-17A^+^ Th17 response (**Supplementary Figure 2**). These results demonstrate the upregulation of the effector CD4^+^ T-cell response in colorectal carcinogenesis.

### ILCs shift during the carcinoma development

**Figure 4:**
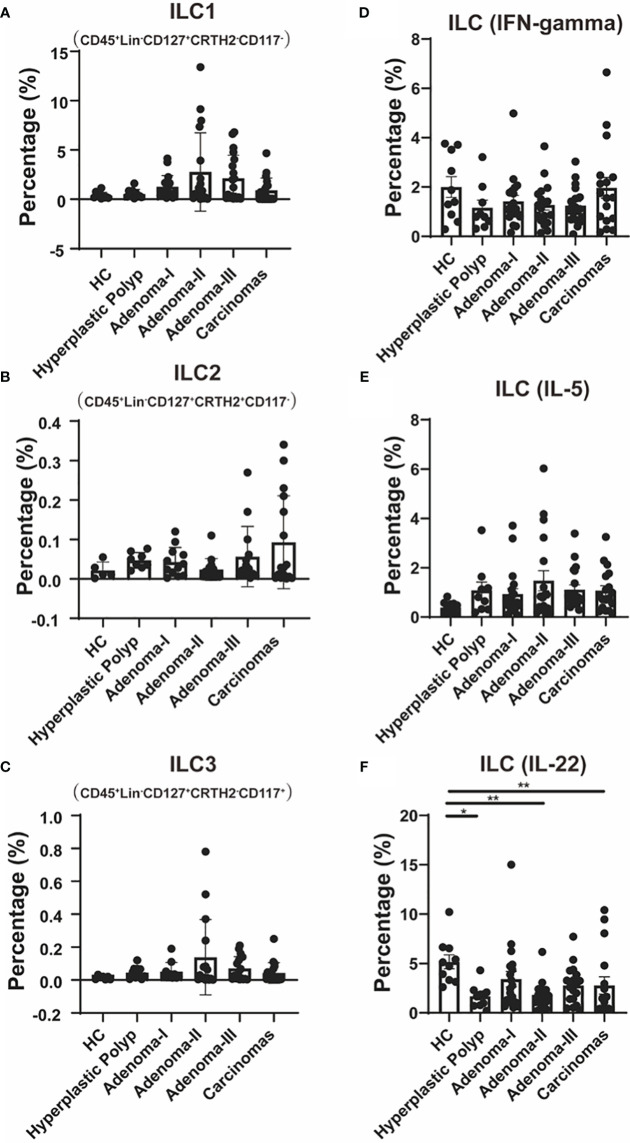
The change of ILC cell populations during a cascade of colonic lesions. **(A–C)** Flow cytometry quantification of the frequency of Lin^-^CD127^+^CRTH2^-^CD117^-^ ILC1 cells **(A)**, Lin^-^CD127^+^CRTH2^+^CD117^-^ ILC2 cells **(B)**, and Lin^-^CD127^+^CRTH2^-^CD117^+^ ILC3 cells **(C)** in CD45^+^ PBMCs during a cascade of colonic lesions. **(D–F)** Flow cytometry quantification of the frequency of Lin^−^CD127^+^IFN-gamma^+^ cells **(D)**, Lin^−^CD127^+^IL-5^+^ cells **(E)**, and Lin^−^CD127^+^IL-22^+^ cells **(F)** in CD45^+^ PBMCs during a cascade of colonic lesions. Each dot represents one donor. HC, healthy control. Error bars represent the SEM. *P < 0.05; **P < 0.01 (ANOVA or non-parametric test as appropriate). *n* = 10–19 in each group.

ILC subsets were further clarified, and gating was performed as previously described (**Supplementary Figure 3**). The CD45^+^Lin^−^CD127^+^CRTH2^−^CD117^−^ ILC1 cell frequency had no significant differences between the control and HPs (**Figure 4A**). The median level of ILC1s showed an increased and then a decreased tendency during the carcinoma development process. We also analyzed circulating CD45^+^Lin^−^CD127^+^CRTH2^+^CD117^−^ ILC2s in patients with colonic diseases. Although an upward trend was found in the disease group compared with the HC group, no significant differences were detected in the frequency of ILC2 cells among all groups (**Figure 4B**).

Moreover, CD45^+^Lin^−^CD127^+^CRTH2^−^CD117^+^ ILC3 was also analyzed in patients with a cascade of colonic disease, which also showed non-significantly increased frequency in colorectal precancerous lesions and CRC (**Figure 4C**).

We further demonstrated the key cytokine production in ILCs to identify their function variation during intestinal carcinogenesis. As shown in **Figure 4D**, the levels of IFN-gamma, mainly produced by ILC1, showed no difference among the groups.

We also measured IL-5 levels, primarily produced by ILC2, and found that there was also no difference between the groups (**Figure 4E**). The IL-22, which is primarily produced by ILC3 and plays a protective role in the intestinal mucosal barrier (30), was further measured to further confirm the involvement of ILC3 in colonic disease development. Compared with the control, IL-22 levels were lower in HPs (4.93 vs. 1.76, *P* = 0.0163), adenoma grade II (4.93 vs. 1.66, *P* = 0.0059), and CRC (4.93 vs. 1.28, *P* = 0.0095) (**Figure 4F**). These results suggest that ILC3s’ function, especially IL-22 production, might be involved in both premalignant lesions, such as adenomas and HPs, and CRC.

### Regulatory T-cell proportions increase from adenomas to carcinomas

**Figure 5:**
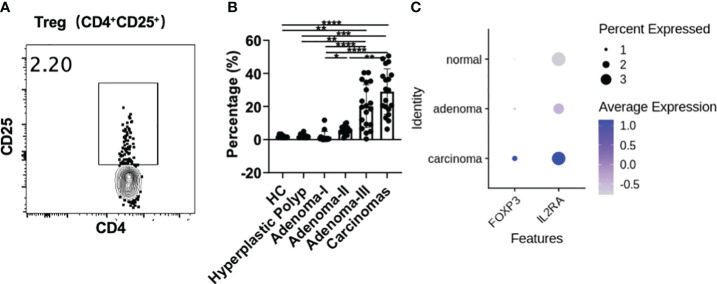
Treg cell analysis during a cascade of colonic lesions. **(A)** The representative flow plots about the gating strategy of CD25^+^ Treg cells in CD4^+^ T cells during a cascade of colonic lesions. **(B)** Flow cytometry quantification of the frequency of CD4^+^CD25^+^ Treg cells in CD4^+^ T cells. Each dot represents one donor. HC, healthy control. Error bars represent the SEM. *P < 0.05; **P < 0.01; ***P < 0.001; ****P < 0.0001 (ANOVA or non-parametric test as appropriate). *n* = 10–19 in each group. **(C)** *IL-2RA* (*CD25*) and *FOXP3* expression analysis in the tissue-derived CD4^+^ T cells among different colonic lesions by analyzing the single-cell RNA-sequencing data (GSE161277). The color depth represents the average expression, and the size of the dots represents the percentage expressed.

The CD4^+^CD25^+^ regulatory T cell (Treg) percentage in PBMCs was investigated in all groups to further assess the frequency change in Tregs in colonic precancerous lesions and CRC (**Figures 5A, B**). The frequency of peripheral CD4^+^CD25^+^ Treg cells was comparable between the control and HP groups. However, compared with the control, a significantly increased percentage of circulating CD4^+^CD25^+^ Tregs was detected in the adenoma grade III (1.56 vs. 19.6, *P* = 0.0032) and CRC groups (1.56 vs. 29.9, *P* < 0.0001). The frequency of CD4^+^CD25^+^ Treg cells gradually increased from early to advanced adenomas and finally to CRC, suggesting that Treg cells increased to exert immunosuppressive effects as adenomas progressed toward CRC (grade I vs. grade II, 0.63 vs. 5.30, *P* = 0.0400; grade I vs. grade III, 0.63 vs. 19.6, *P* < 0.0001; grade I vs. CRC, 0.63 vs. 29.9, *P* < 0.0001; grade II vs. CRC, 5.30 vs. 5.30, *P* = 0.0059). Moreover, CD4^+^CD25^+^ Treg cells were decreased in HPs compared with grade III adenoma (1.96 vs. 19.6, *P* = 0.0097) or CRC (1.96 vs. 29.9, *P* = 0.0001). There was no difference between HPs and adenoma grade I or II.

Furthermore, single-cell RNA-sequencing data showed that the expression of *IL-2RA* (*CD25*) and *FOXP3*, the key markers of Treg cells, was increased from adenoma to carcinoma in the tissue-derived CD4^+^ T cells (**Figure 5C**), which is consistent with the changes in peripheral Treg cells. This result further confirms the involvement of Tregs in colonic premalignant lesions and CRC.

### T follicular helper cell response is downregulated during colorectal carcinogenesis

**Figure 6:**
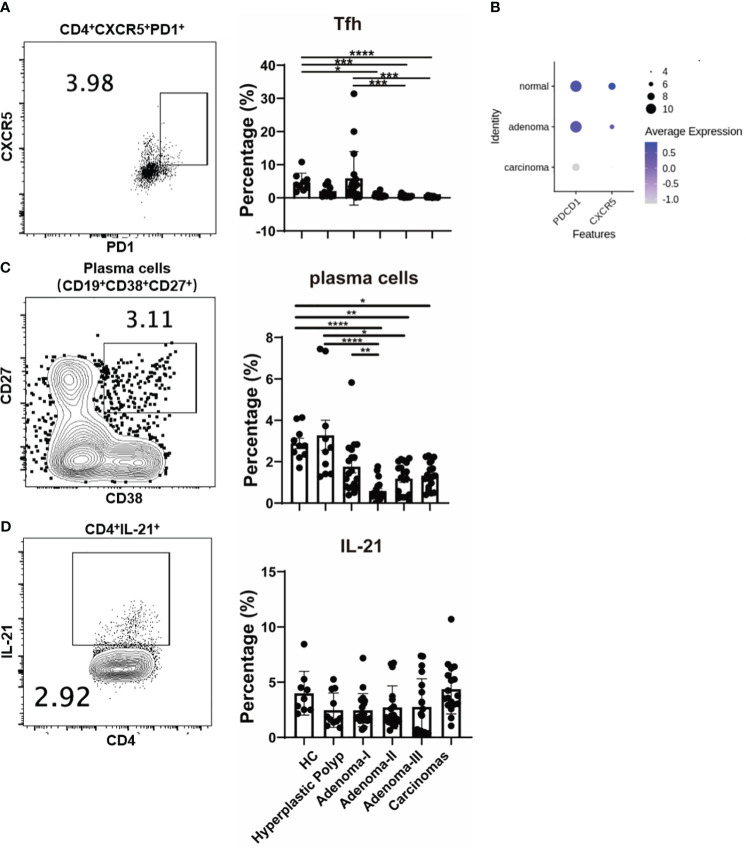
The change of Tfh cells during a cascade of colonic lesions. **(A)** The FACS staining (left) and frequency quantification (right) analysis of CD4^+^CXCR5^+^ PD1^+^ cells in CD4^+^ T cells during a cascade of colonic lesions. **(B)** The expression analysis of *PDCD1* and *CXCR5* in the tissue-derived CD4^+^ T cells among different colonic lesions by analyzing the single-cell RNA-sequencing data (GSE161277). The color depth represents the average expression, and the size of the dots represents the percentage expressed. **(C)** The FACS staining (left) and frequency quantification (right) analysis of CD19^+^CD38^+^CD27^+^ plasma cells during a cascade of colonic lesions. **(D)** The FACS staining (left) and frequency quantification (right) analysis of IL-21^+^ cells in CD4^+^ T cells during a cascade of colonic lesions. Each dot represents one donor. HC, healthy control. Error bars represent the SEM. *P < 0.05; **P < 0.01; ***P < 0.001; ****P < 0.0001 (ANOVA or non-parametric test as appropriate). *n* = 10–19 in each group.

We further analyzed the T follicular helper (Tfh) response, and CD4^+^PD1^+^CXCR5^+^ cells were significantly decreased in adenoma grade II (3.95 vs. 0.63, *P* = 0.0157), grade III (3.95 vs. 0.26, *P* = 0.0002), and CRC (3.95 vs. 0.16, *P* < 0.0001) compared with the control (**Figure 6A**). Moreover, the percentage of CD4^+^PD1^+^CXCR5^+^ cells decreased from adenoma grade I to III and even in CRC (**Figure 6A**), primarily attributed to the change in programmed cell death protein 1 (PD1) but not CXCR5 (**Supplementary Figures 4A, B**). In addition, the expression of *PDCD1* and *CXCR5* in the tissue-derived CD4^+^ T cells among different colonic lesions was analyzed using the single-cell RNA-sequencing data (GSE161277). As shown in **Figure 6B**, the Tfh cell markers *PDCD1* and *CXCR5* were decreased during the carcinoma progression, which is consistent with the change of t peripheral Tfh population. Furthermore, the percentage of antibody-secreting CD19^+^CD38^+^CD27^+^ plasma cells also decreased in the adenoma grade II group (2.775 vs. 0.405, *P* < 0.0001), adenoma grade III group (2.775 vs. 1.230, *P* = 0.0015), and CRC group (2.775 vs. 1.280, *P* = 0.0155) compared with that in the control group (**Figure 6C**). However, IL-21^+^ production in CD4^+^ cells, the key cytokine of Tfh cells, showed no significant differences among the groups (**Figure 6D**). These results suggested a potential role of Tfh cells in both precancerous lesions and CRC.

## Discussion

The ability of precancerous lesions to progress to cancer is related to the intrinsic phenotype and the contributions of the immune response. Therefore, an in-depth investigation of the immunological characteristics of colorectal carcinoma progression is crucial for understanding the mechanisms underlying CRC development. Tumor occurrence and development often result in systemic immune disturbances and alterations in peripheral immune cells through tissue communication with peripheral blood (21–24). Therefore, monitoring disease development through the peripheral immune response may be a better option. Dynamic changes in the peripheral immune profile, from colonic precancerous lesions to CRC lesions, have not been reported. In this study, we demonstrated the immune landscape of the CRC development process, including changes in Th cells, ILC cells, and key cytokines that play critical roles in maintaining intestinal homeostasis.

Cytokines are a class of small molecular proteins or peptides with biological activities that can be divided into pro- and anti-inflammatory cytokines. The cytokines in our study exhibited non-significant subtle changes. IFN-γ, TNF-α, and IL-2 enhance cytotoxic and apoptotic effects in response to colon adenomas. IL-22 plays both protective and pathogenic roles in inflammation. At an early stage, IL-22 is protective, helps maintain barrier integrity, and reduces inflammation and tumors. However, during wound repair in the epithelium, IL-22 promotes tumor development (31). Our results were inconsistent with those of previous studies, and no changes were detected in the plasma cytokine spectrum, indicating that their ability to detect lesions might be limited.

We comprehensively analyzed lymphocyte profiles in premalignant and colorectal tumors. ILCs are central innate immune mediators in both gastrointestinal homeostasis and inflammatory pathologies (15) and exhibit striking similarities to the heterogeneity in CD4^+^ T helper cells (14, 32). To our knowledge, our results are the first to demonstrate the characteristics of Th and ILC subsets in HPs. Though effector Th response increased during the carcinoma process, the ILC-derived IL-22 production was downregulated in the HP, adenoma, and CRC groups. The immunological characteristics of HP, adenoma, and CRC are generally consistent with the tissue-derived single-cell RNA-seq data from previously reported studies (20, 27). Interestingly, the peripheral CD4^+^ T cells were contrary to the results of tumor-derived CD4^+^ T cells in terms of their surface molecules. This might be due to the differences in methodology because the peripheral CD4^+^ T cells were defined with surface molecules at the protein level while the tumor-derived CD4^+^ T cells’ surface molecules were analyzed at the RNA level. Moreover, the differences in localization might also contribute to that. The upregulation of these chemokines in the tumor-derived CD4^+^ T cells was very likely associated with the migration toward tumor tissues. Considering the easily obtainable and non-invasive characteristics of peripheral blood samples, the circulating Th cell analysis might be used to monitor CRC development and discover key functional cell subpopulations during CRC carcinogenesis.

Maintaining the immune balance is critical for antitumor immunity. Foxp3^+^ regulatory T cells secrete immunomodulatory cytokines and cytolytic molecules that regulate immune responses (33). Besides the effect of Th subsets, suppressive Treg cells also play an essential role in the tumor immune response. Elevated Treg cells are associated with promoting tumor development, immunotherapy failure, and poorer prognosis in CRC (34), suggesting that the immune balance is critical in antitumor immunity. In terms of tumor prognosis, a high number of Tregs are correlated with poor patient survival. Foxp3^+^ Treg accumulation in the tumor microenvironment is an early event along the carcinoma development and may play a role in initiating CRC (35). Our study confirmed the upregulation of peripheral Tregs during carcinoma progression and showed a progressive increase from adenoma grade I to CRC. However, Treg activation induced CD25 upregulation in CD4^+^ conventional T cells (36, 37). Therefore, we cannot exclude the involvement of activated T cells in the CD4^+^CD25^+^ population. We also investigated the changes in different effector CD4^+^ cell subsets, and these populations did not show dramatic changes as the CD4^+^CD25^+^ populations. Thus, we speculated that the changes in CD4^+^CD25^+^ cells may be mainly attributed to Treg cells. In addition, the tendency of peripheral Treg cells to increase during CRC development was consistent with the results from colonic tissues, indicating the potential use of peripheral Treg cells to monitor CRC progression.

As a critical CD4^+^ T-cell subset, Tfh cells primarily function by interacting with B cells and are essential for guiding immunoglobulin isotype switching, affinity maturation, and memory- and antibody-secreting B-cell differentiation (38). Tfh cells help B cells during effective antibody-mediated immune responses (38, 39). Recently, the role of Tfh cells in the antitumor immune response has attracted increasing attention (37, 39). Tfh indirectly enhances antitumor immunity mediated by CD8^+^ T cells by secreting IL-21 (40). Furthermore, the specific intestinal bacterium *Helicobacter hepaticus* promotes Tfh-associated antitumor immunity in the colon (37). However, the involvement of Tfh cells and related antibody responses in CRC development is unclear. Our results further demonstrate that Tfh cells and related plasma cell populations decrease with disease progression, implying that Tfh cells are involved in precancerous and CRC stages.

However, our study has limitations, such as a small cohort of unpaired individuals. Considering this limitation and to further confirm the tissue specificity of the changed cell populations, we verified our PBMC results using published single-cell RNA-seq data by detecting the expression levels of Th cell subset markers in CD4^+^ T cells from tissues. Tissue results showed the same variation tendency as that of the PBMCs. This finding verified our PBMC findings and further indicated that the changes in Th subsets may be primarily due to colorectal lesions. However, another model is needed to clarify whether this is a sequential effect, such as using paired tissue samples or collecting PBMCs at different pathological stages from the same patients. Moreover, the male/female ratios of the samples were inconsistent between the groups. The male/female ratio was lower in the adenoma group than in the control/CRC group. The small sample size also caused a potential bias during the short collection period, which may have confounded the results. Sexual dimorphisms have been described in innate and adaptive immune systems. Significantly elevated frequencies of Treg cells were reported in the peripheral blood of young postpubertal cisgender men compared with similarly aged cisgender women. Thus, sex chromosomes and hormones may drive changes in Treg cell frequency and function, and young postpubertal men have a more anti-inflammatory Treg cell profile than women (41). A higher incidence of CRC is observed in men than in women (42). Although the control group had a high male ratio in our study, the percentage of Tregs was still lower than that in the adenoma and carcinoma groups. This finding indicates that changes in Tregs in colonic precancerous lesions and CRC may result from pathological changes. As for Tfh cells, no studies have reported the influence of sex on Tfh cells in colonic precancerous lesions and CRC. However, the relationship between elevated levels of circulating Tfh cells and female-biased autoimmune diseases has been verified (43). Accordingly, the decreased Tfh percentage may not be due to sex but to the lower female ratio in the control group. Since the exact influence of sex hormones on the immune phenotype during tumor development remains unclear, the effect of sex bias should be considered. Lastly, because ILC2s can also express CD117 (44), some ILC2s might be excluded from the analysis.

In summary, we analyzed the immunological profile characteristics and demonstrated the involvement of Th subsets, especially Treg and Tfh cell populations, during colorectal carcinogenesis. Our study is the first to demonstrate the lymphocyte profiles of HPs and the CRC development process by analyzing circulating PBMCs and the production of key cytokines. Further exploration of their functions is required to develop a precise treatment.

## Conclusion

We analyzed circulating ILCs and adaptive T lymphocyte subtypes in colorectal carcinogenesis. We revealed the involvement of Th subsets, especially Treg and Tfh cells, in CRC development and clarified the immunological characteristics of HPs.

These findings significantly enhance our understanding of the immune mechanisms underlying CRC and its precancerous lesions. Further investigation of the Treg and Tfh cells’ function in colorectal disease development will provide potential therapeutic targets for monitoring and preventing CRC development. Considering the easily obtainable and non-invasive characteristics of peripheral blood samples, demonstrating the change of peripheral functional cell subpopulations, like Treg and Tfh cells, during CRC carcinogenesis might provide novel target cells for the early screening of CRC patients.

## Data availability statement

The raw data supporting the conclusions of this article will be made available by the authors, without undue reservation.

## Ethics statement

The studies involving humans were approved by the Peking University Third Hospital Medical Science Research Ethics Committee. The studies were conducted in accordance with the local legislation and institutional requirements. The human samples used in this study were acquired from a by-product of routine care or industry. Written informed consent for participation was not required from the participants or the participants’ legal guardians/next of kin in accordance with the national legislation and institutional requirements.

## Author contributions

QM: Data curation, Methodology, Software, Validation, Visualization, Writing – original draft. YZ: Data curation, Writing – original draft. MX: Writing – review & editing. PW: Writing – review & editing. JL: Writing – original draft. RC: Writing – original draft. WF: Conceptualization, Formal analysis, Funding acquisition, Supervision, Writing – review & editing. SD: Funding acquisition, Project administration, Writing – review & editing.
