# Supplementary material for: Increased circulating regulatory T cells and decreased follicular T helper cells are associated with colorectal carcinogenesis
Source: Front Immunol. 2024 Jan 26;15:1287632. doi: 10.3389/fimmu.2024.1287632 (PMC10853383; doi:10.3389/fimmu.2024.1287632)
Supplement: Supplementary file 1 [file DataSheet_1.docx]

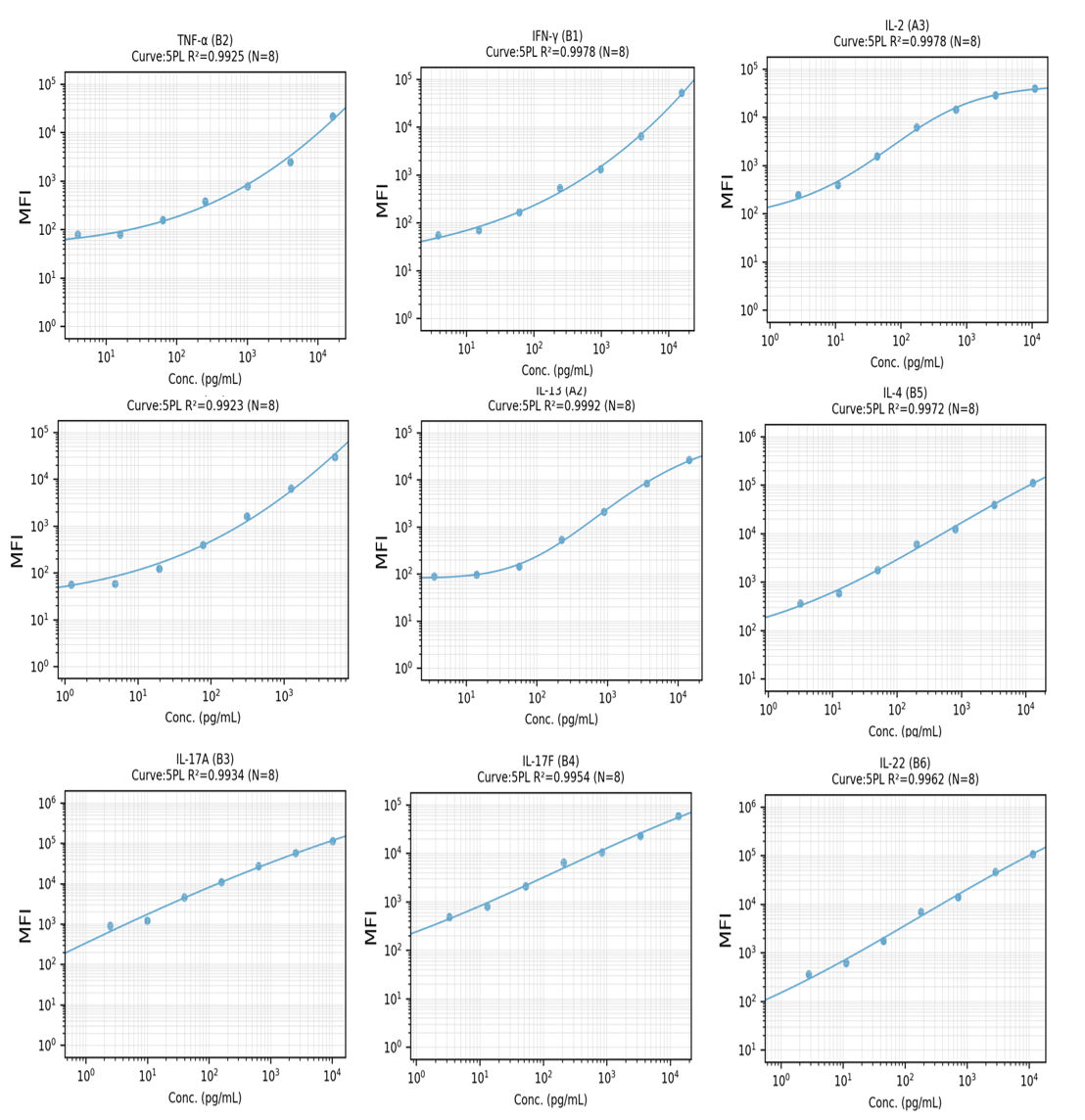


**Supplemental Figure 1**

Standard curve of plasma cytokines using BCA. Horizontal axis represents concentration; vertical axis represents maximum fluorescence intensity (MFI). n = 10-19 in each group.


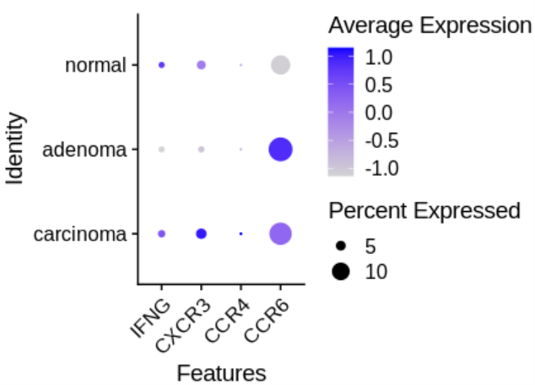


**Supplemental Figure 2**

*IFN-gamma，CXCR3* (Th1 cell surface marker), *CCR4* (Th2 cell surface marker), and *CCR6 (*Th17 cell surface marker) expression analysis in the tissue derived CD4^+^T cells among different colonic lesions by analyzing the single-cell RNA sequencing data (GSE161277). The color depth represents average expression, and the size of the dots represents percentage expressed.


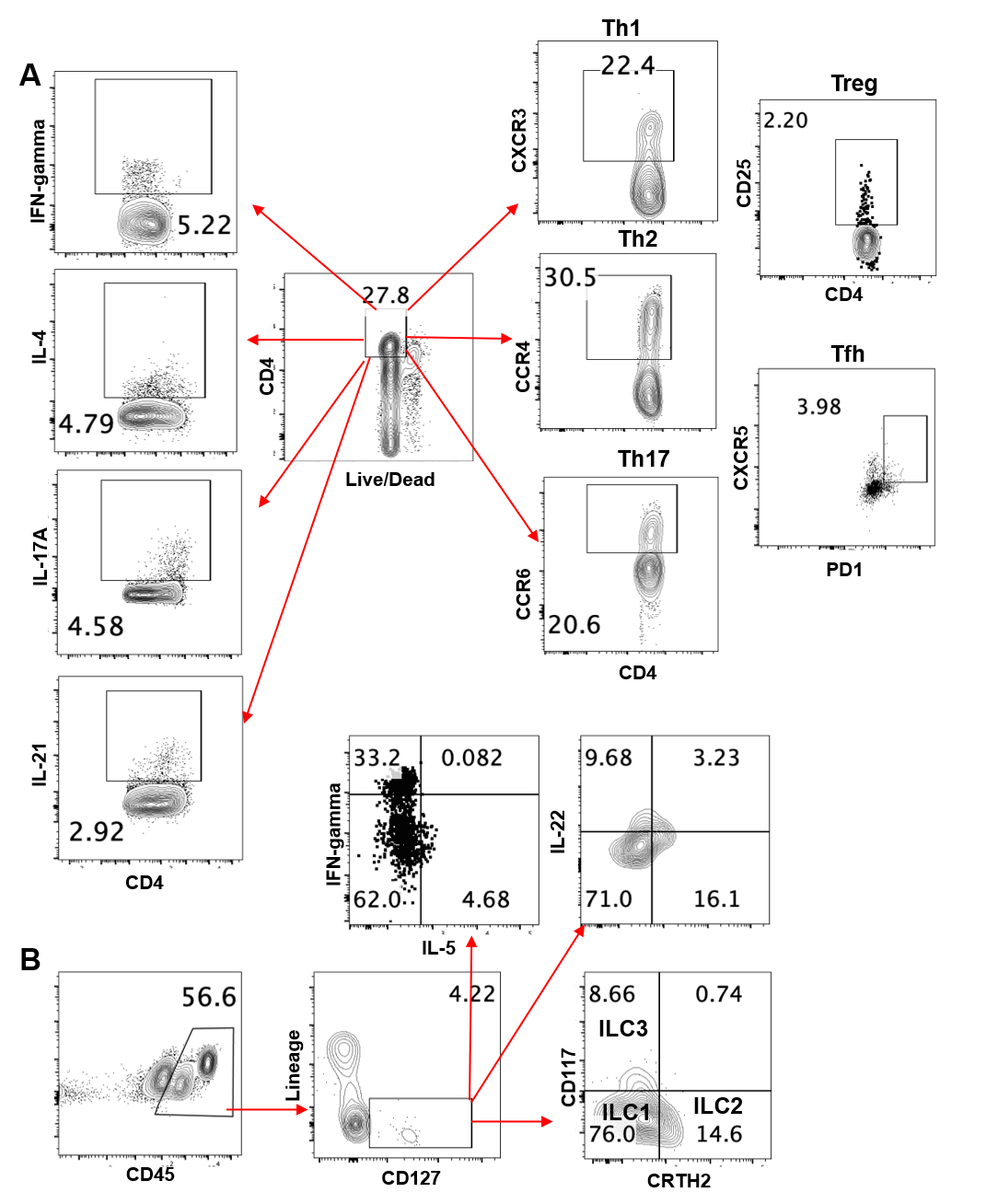


**Supplemental Figure 3 Gating strategy of human Th and ILC cells subsets**

(A)Th subsets were annotated as Th1 cell (CXCR3^+^), Th2 cell (CCR4^+^), Th17 cell (CCR6^+^), Treg cells (CD4^+^CD25^+^), and Tfh cells (CXCR5^+^PD1^+^) in CD4^+^ live cells. The respective key functional cytokines were gated as IFN-gamma^+^ (for Th1 cells), IL-4 ^+^ (for Th2 cells), IL-17A ^+^ (for Th17 cells), and IL-21 ^+^ (for Tfh cells). (B) ILCs were gated as CD45^+^ and lineage negative, CD127^+^ and further divided by expression of CRTH2 (ILC2) or CD117 (ILC3) or as lacking expression of both markers (ILC1).


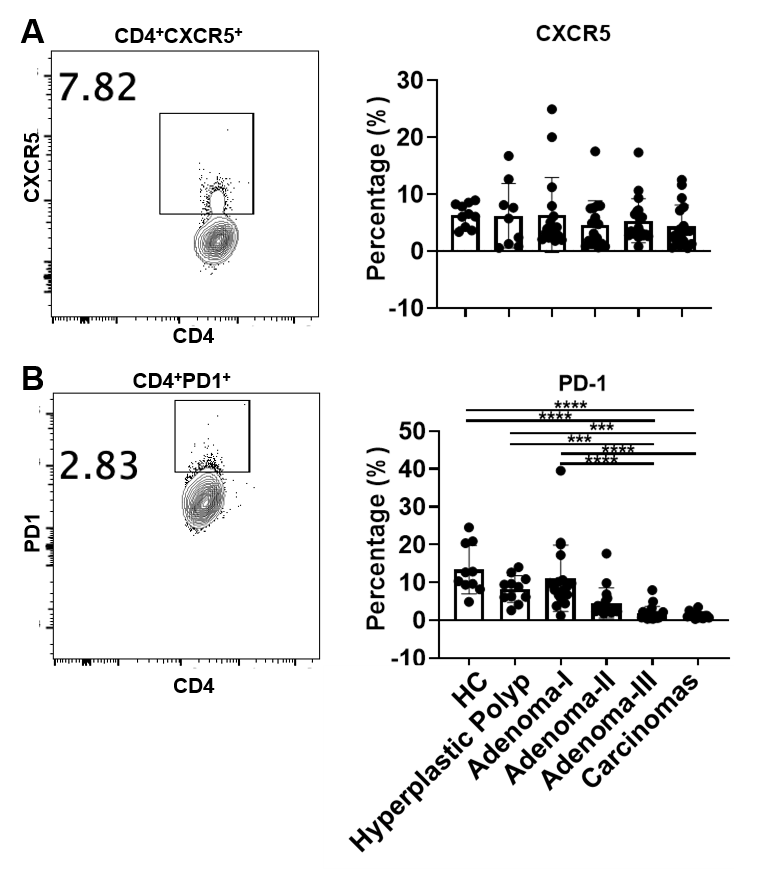


**Supplemental Figure 4**

The representative flow plots (left) and frequencies of CXCR5^+^ cells (A) and PD1^+^ cells (B) in CD4^+^T cells during a cascade of colonic lesions. n = 10-19 in each group. Error bars represent the SEM. *** P < 0.001; **** P < 0.0001 (ANOVA or non-parametric test as appropriate). n = 10-19 in each group.

**Supplemental Table 1 Key resources table**

|  | Source | Clone | Concentration per cell |
| --- | --- | --- | --- |
| APC anti-human CD24 Antibody | Biolegend | ML5 | 5 µl per million cells |
| PE/Cyanine7 anti-human CD27 Antibody | Biolegend | M-T271 | 5 µl per million cells |
| FITC anti-human CD38 Antibody | Biolegend | HIT2 | 5 µl per million cells |
| APC/Cyanine7 anti-human CD45 | Biolegend | HI30 | 5 µl per million cells |
| APC anti-human CD117 (c-kit) | Biolegend | 104D2 | 5 µl per million cells |
| PE anti-human CD127 (IL-7Rα) | Biolegend | A019D5 | 5 µl per million cells |
| PE/Cy7 anti-human CD294 (CRTH2) | Biolegend | BM16 | 5 µl per million cells |
| APC/Cy7 anti-human CD4 | Biolegend | RPA-T4 | 5 µl per million cells |
| PerCP anti-human CD183 (CXCR3) | Biolegend | G025H7 | 5 µl per million cells |
| PE/Cy7 anti-human CD194 (CCR4) | Biolegend | L291H4 | 5 µl per million cells |
| APC anti-human CD196 (CCR6) | Biolegend | G034E3 | 5 µl per million cells |
| PE anti-human CD25 | Biolegend | BC96 | 5 µl per million cells |
| PerCP/Cyanine5.5 anti-human IL-21 | Biolegend | 3A3-N2 | 5 µl per million cells |
| FITC anti-human CD19 | Biolegend | HIB19 | 5 µl per million cells |
| Brilliant Violet 421™ anti-mouse/human IL-5 Antibody | Biolegend | TRFK5 | 5 µl per million cells |
| Gata-3 Monoclonal Antibody (TWAJ), PE | eBioscience | TWAJ | 5 µl per million cells |
| Brilliant Violet 421™ anti-T-bet Antibody | Biolegend | 4B10 | 5 µl per million cells |
| APC anti-human IFN-γ Antibody | Biolegend | 4S.B3 | 5 µl per million cells |
| IL-17A Monoclonal Antibody | eBiosciences | 64DEC17 | 5 µl per million cells |
| CD279 (PD-1) Monoclonal Antibody | eBiosciences | J105 | 5 µl per million cells |
| ROR gamma (t) Monoclonal Antibody | eBiosciences | AFKJS-9 | 5 µl per million cells |
| Horizon™ BV421 Rat Anti-Human CXCR5 (CD185) | BD Biosciences | RF8B2 | 5 µl per million cells |
| FITC Rat Anti-Human IL-4 | BD Biosciences | MP4-25D2 | 0.5µg per million cells |
